# Letter to the Editor on “Effectiveness of early cardiorespiratory rehabilitation combined with melatonin supplementation during the inpatient period following acute myocardial infarction.”

**DOI:** 10.1016/j.arrct.2025.100476

**Published:** 2025-06-03

**Authors:** Amine Ghram, Helmi Ben Saad

**Affiliations:** Cardiac Rehabilitation Department, Heart Hospital, Hamad Medical Corporation, Doha, Qatar.; Research Laboratory “Heart Failure, LR12SP09”, Hospital Farhat Hached of Sousse, Sousse, Tunisia.; Healthy Living for Pandemic Event Protection (HL-PIVOT) Network, Chicago, IL.; Research Laboratory “Heart Failure, LR12SP09”, Hospital Farhat Hached of Sousse, Sousse, Tunisia.; Laboratory of Physiology and Functional Explorations, Farhat Hached Hospital, Sousse, Tunisia.; Laboratory of Physiology, Faculty of Medicine of Sousse, University of Sousse, Sousse, Tunisia.

Dear Editor,

I read with great interest the article by Hbaieb et al,^1^ which explores an innovative combination of early cardiorespiratory rehabilitation and melatonin supplementation in patients with postacute myocardial infarction. This is interesting because the aforementioned combination is justified by its complementary physiological benefits, the ability to intervene during a critical window of vulnerability, and its potential to modulate key pathophysiological mechanisms after acute myocardial infarction. While we commend the authors for investigating this novel combination, 2 methodological concerns related to the sample size calculation and the 6-minute walk test (6MWT) merit attention.

Concerning the sample size calculation, where the primary endpoint was the 6-minute walk distance (6MWD), the authors opted for a statistical formula based on effect size and reported that a sample of 45 patients (ie, 3 groups of 15 each) had adequate power to detect statistical effects. It was better to calculate the sample size based on the minimal clinically important difference (MCID) for a change in the 6MWD in adults with chronic pathology.^2^ For instance, a 2016 systematic review comprising 6 studies, including individuals with cardiorespiratory chronic conditions and adults with a fear of falling, reported that the MCID for which the area under the receiver operating characteristic curve was at least 0.70 ranged from 14.0 to 30.5 m.^2^ Moreover, the 6MWT international guidelines^3^ reported that in adults with chronic diseases, the 6MWD MCID lies between 25 and 33 m. Considering a 6MWD MCID of 30 m, a power (1−β) of 80% (typically paired with α=.05), and analysis of variance as the primary analysis, a sample size of 35 per group (105 total) is needed. Using an insufficient number of participants, as done in the Tunisian study,^1^ may result in lower precision of the results.

Concerning the 6MWT, Hbaieb et al^1^ omitted to report which 6MWT guideline was applied. First, because the study was conducted in 2022 and 2023, it would be beneficial to inform readers that the most up-to-date international guideline was used.^3^ Second, the expression of the main outcome (ie, 6MWD) in absolute value only and the lack of standardization according to participants’ characteristics (eg, sex, anthropometric data) could lead to misinterpretation.^4^ The standardized 6MWD, by applying local 6MWD norms, for example, allows for a more objective comparison between diverse groups.^4^ Several studies have shown that the percentage predicted 6MWD correlates better with outcomes such as mortality, hospitalization, and quality of life than the absolute distance.^5^ Third, the retained definition of impaired functional capacity (ie, 6MWD <450 m), based on a reference that includes patients with atrial fibrillation (their reference: number 29), is a source of confusion. It was better to consider each 6MWD lower than the lower limit of the normal range as an abnormal 6MWD and, therefore, show impaired functional capacity.^4^

In conclusion, choosing the right sample size is essential because it guarantees a representative sample for identifying statistical significance and helps prevent insufficient power to detect statistical effects. Furthermore, we advise defining impaired functional capacity using a more precise method, such as individualized lower bounds of normal, and standardizing the 6MWD using local norms in order to effectively convey it.

## Disclosure

The investigators have no financial or nonfinancial disclosures to make in relation to this project.
